# PlantPathNet: a novel network based on cross-layer feature integration for plant disease classification

**DOI:** 10.3389/fpls.2026.1783576

**Published:** 2026-04-15

**Authors:** Demet Parlak Sönmez, Şafak Kılıç

**Affiliations:** 1 Department of Software Engineering, Faculty of Engineering, Architecture and Design, Kayseri University, Kayseri, Türkiye; 2 School of Computer Science, University of Nottingham, Nottingham, United Kingdom

**Keywords:** attention mechanism, class imbalance, feature integration, fully convolutional networks, HSV color space, leaf image classification, loss functions, plant disease diagnosis

## Abstract

**Introduction:**

Reliable identification of plant diseases from leaf images is essential for effective crop monitoring and the prevention of yield deterioration. With the growing adoption of deep learning in agricultural applications, convolutional neural network–based classifiers have demonstrated notable success in visual plant disease recognition.

**Methods:**

In this study, we propose *PlantPathNet*, a purpose-built deep learning architecture for plant disease classification. Input images are transformed from the RGB to the HSV color space to enhance the representation of disease-related visual features. A novel Cross-layer Feature Integration Module (CFIM) is introduced to effectively aggregate discriminative features across multiple network depths. Additionally, an efficient channel attention mechanism based on ECANet is incorporated to emphasize disease-relevant representations. The model is optimized using a composite loss function combining modified softmax loss and center loss to address class imbalance and improve feature separability.

**Results:**

Extensive experiments conducted on the PlantVillage dataset demonstrate that PlantPathNet outperforms several state-of-the-art models, including ResNet-50, Inception-V3, DenseNet121, VGG16, and Vision Transformer-based approaches. The proposed model achieves an overall accuracy of 99.57%, precision of 99.52%, recall of 99.54%, F1-score of 99.53%, and an AUROC of 99.84%.

**Discussion:**

The results indicate that the integration of HSV-based preprocessing, CFIM, and channel attention significantly enhances classification performance. The proposed framework provides a robust and efficient solution for automated plant disease diagnosis and has strong potential for real-world agricultural applications.

## 1 Introduction

Agriculture constitutes a cornerstone of the global economy; however, plant diseases persist as a major challenge, accounting for an estimated 20% to 40% of annual global crop losses (Nazarov et al., 2020). Conventional detection practices predominantly depend on manual visual assessments, which are inherently time-consuming, subjective, and often insufficient for early-stage diagnosis, particularly in developing regions (Moshou et al., 2005). Recent progress in computer vision has opened new avenues for automated systems that hold significant potential to transform these conventional practices (Naikwadi and Amoda, 2013).

Deep learning methods, particularly Convolutional Neural Networks (CNNs), are well-suited for plant disease detection due to their hierarchical representation learning capability. These architectures automatically map raw input data into abstract representations, capturing both low-level visual patterns and high-level semantic information (Han et al., 2023; Shoaib et al., 2023). Various CNN-based frameworks, such as ResNet and Inception, have already reported encouraging results across multiple crop types (He et al., 2015; Huang et al., 2018).

In this work, we aim to tackle the aforementioned challenges by leveraging the PlantVillage dataset to design and systematically evaluate efficient deep learning–based frameworks for plant disease detection. By integrating advanced image preprocessing strategies with carefully optimized neural network architectures, the proposed approach seeks to deliver accurate, timely, and computationally efficient disease diagnosis. Such a framework is expected to facilitate practical deployment in real-world agricultural settings, thereby supporting improved crop management practices and contributing to enhanced agricultural productivity and global food security. However, a significant challenge in this domain is the gap between laboratory-curated datasets and unpredictable field conditions. Most existing models, while accurate on benchmark data, struggle with the visual noise, varying lighting, and complex backgrounds found in real-world orchards (Mohanty et al., 2016; Barbedo, 2018). PlantPathNet is specifically engineered to bridge this gap by utilizing HSV transformation to isolate chromatic disease cues from environmental distractions.

### 1.1 Our contributions

Motivated by the aforementioned challenges, this study introduces *PlantPathNet*, a novel deep learning framework for plant disease diagnosis developed and evaluated on the PlantVillage dataset. The proposed architecture is specifically designed to enhance disease recognition performance through effective cross-layer feature fusion and attention-driven representation learning. By integrating targeted architectural and training strategies, the model aims to achieve high classification accuracy while maintaining computational efficiency. The main contributions of this work are summarized as follows:

- We introduce *PlantPathNet*, a novel deep learning architecture that incorporates a Cross-layer Feature Integration Module (CFIM) to effectively fuse representations from multiple convolutional layers, enabling improved detection of subtle and fine-grained disease patterns in plant leaf images.
- A color space transformation from RGB to HSV is employed to enhance the representation of chromatic variations and textural cues associated with plant diseases, leading to more discriminative feature extraction compared to conventional RGB-based inputs.
- An Adaptive Attention Mechanism (AAM) is integrated into the network to selectively emphasize disease-relevant regions, thereby strengthening the model’s ability to focus on informative visual features while suppressing background noise.
- A balanced optimization strategy is proposed by combining weighted softmax loss with center loss, which effectively mitigates class imbalance while simultaneously improving intra-class compactness and inter-class separability.
- Extensive experimental evaluations on the PlantVillage dataset demonstrate that the proposed framework consistently outperforms several state-of-the-art models, including ResNet-50, Inception-V3, DenseNet121, VGG16, and representative Vision Transformer architectures. The proposed PlantPathNet achieves a peak accuracy of 99.57% on the Plant Village dataset, consistently outperforming established baselines across all evaluated metrics.
- A statistical significance analysis is conducted to validate the robustness and reliability of the observed performance improvements over existing deep learning approaches.

The remainder of this paper is organized as follows. Section 2 reviews related studies on deep learning–based plant disease detection. Section 3 details the proposed PlantPathNet architecture, along with the dataset description and preprocessing procedures. Experimental results and comparative performance analyses are presented in Section 4. Finally, Section 5 concludes the paper and discusses potential directions for future research.

## 2 Related work

Early and reliable identification of plant diseases is of paramount importance for improving agricultural productivity and safeguarding global food security. In recent years, substantial progress in computer vision and deep learning has enabled the development of automated frameworks capable of accurately recognizing plant diseases from visual data.

Historically, plant disease diagnosis and classification have largely depended on manual visual inspections performed by experienced farmers or plant pathologists. Although effective to some extent, this practice is inherently subjective, labor-intensive, and unsuitable for large-scale or real-time monitoring scenarios (Barure et al., 2020). To overcome these limitations, machine learning–based approaches have been increasingly explored, with a variety of algorithms demonstrating promising performance in automated plant disease detection and classification tasks.

Maniyath et al. (2018) investigated the use of a Random Forest classifier for papaya leaf disease detection and reported an accuracy of 70.14%. Komala (2021) applied a combination of image preprocessing and filter-based feature selection techniques, achieving an accuracy of 95% using a KNN classifier. Similarly, Abdu et al. (2020) conducted a comparative analysis between Support Vector Machine (SVM) models and deep learning–based approaches, showing that the SVM classifier attained an accuracy of 95.8%. Although these studies demonstrate the feasibility of conventional machine learning techniques for plant disease diagnosis, their performance is inherently limited by a strong dependence on handcrafted feature extraction.

More recently, deep learning–based methods have led to substantial progress in plant disease detection by enabling end-to-end learning and eliminating the need for manual feature engineering. For instance, Mohanty et al. (2016) reported classification accuracies of up to 99.35% on the PlantVillage dataset using AlexNet and GoogLeNet architectures. Nevertheless, the authors noted a notable performance gap between images captured under controlled laboratory conditions and those obtained in real-world field environments, highlighting ongoing challenges related to generalization and robustness.

CNN-based architectures have been extensively adopted in plant disease detection due to their strong representation learning capability and high classification accuracy. Tan et al. (2021) conducted a comparative evaluation of multiple deep learning models on the PlantVillage tomato dataset, reporting that ResNet34 achieved superior performance with an accuracy of 99.7%, precision of 99.6%, recall of 99.7%, and an F1-score of 99.7%. Likewise, Reddy et al. (2023) proposed a customized PDICNet framework that combines a CNN-based classifier built upon ResNet50 with a modified Red Deer Optimization Algorithm (MRDOA), attaining an accuracy of 99.73% on the PlantVillage dataset.

Beyond disease classification, several studies have focused on disease severity assessment and generalization capabilities. Wspanialy and Moussa (2020) introduced a computer vision system integrating ResNet50 and U-Net architectures to estimate disease severity, with the ability to identify previously unseen disease types, achieving an overall accuracy of 98.7%. In contrast, Fenu and Malloci (2021) investigated the use of EfficientNetB0 for classifying three disease categories across six severity levels, reporting a comparatively lower accuracy of 78.31%, which highlights the increased complexity associated with fine-grained severity estimation tasks.

Transfer learning has proven to be a highly effective strategy for adapting deep learning models pre-trained on large-scale datasets to domain-specific tasks with relatively limited data. Wang et al. (2017) investigated the fine-tuning of several state-of-the-art architectures, including VGG16, VGG19, Inception V3, and ResNet50, for apple leaf disease severity prediction, reporting that the VGG16 model delivered the best performance with an accuracy of 90.4%.

More recently, Anim-Ayeko et al. (2023) introduced a lightweight ResNet9based framework for blight disease detection in potato and tomato leaves. Their approach incorporated leaf shape characteristics, diseased region information, and overall green area analysis, in conjunction with hyperparameter optimization and data augmentation strategies. As a result, the proposed model achieved an accuracy of 99.25%, precision of 99.67%, recall of 99.33%, and an F1-score of 99.33%, demonstrating the effectiveness of optimized transfer learning and lightweight architectures for plant disease detection.

In addition to algorithmic developments, several studies have emphasized practical deployment scenarios for plant disease detection systems. Gandhi et al. (2021) proposed a mobile application based on MobileNetV2 and Siamese network architectures, designed to operate under resource-constrained conditions while achieving an accuracy of 95%. Likewise, Kumbhar et al. (2019) introduced a web-based diagnostic platform for cotton leaf diseases, which not only facilitated disease identification but also provided farmers with recommended preventive and remedial actions.

Despite the demonstrated effectiveness of deep learning–based approaches, a number of critical challenges remain unresolved. Barbedo (2018) examined the influence of dataset size and diversity on classification performance, highlighting that insufficient data variability can significantly limit model generalization. Furthermore, Shi et al. (2023) analyzed the key limitations faced by CNN-based plant disease severity assessment systems in real-world environments and outlined potential research directions to address issues related to robustness, scalability, and practical applicability.

Ngugi et al. (2021) provided a comprehensive review of automated leaf disease recognition systems, documenting a clear transition from handcrafted feature–based methods and shallow classifiers toward deep learning–driven solutions. Similarly, Liu and Wang (2021) systematically analyzed deep learning techniques for plant disease and pest detection, categorizing existing approaches into classification, detection, and segmentation-based frameworks.

Despite the substantial promise of deep learning–based plant disease detection systems, several critical drawbacks persist in existing frameworks. Most current models rely on generic backbones that are not specifically optimized for the unique chromatic and textural features of plant pathologies, leading to performance degradation in complex, real-world field environments (Mohanty et al., 2016; Barbedo, 2018). Furthermore, conventional architectures often suffer from information loss during progressive downsampling and lack effective cross-layer interaction to capture both subtle symptoms and high-level semantic cues simultaneously. *PlantPathNet* overcomes these limitations by integrating a specialized HSV color space transformation to isolate diagnostic chromatic signals and a novel Cross-layer Feature Integration Module (CFIM) that preserves and fuses multi-scale features. By addressing the gap between laboratory-curated data and unpredictable field conditions, the proposed model ensures robust and generalizable performance across diverse environmental scenarios and heterogeneous plant species. Consequently, this study prioritizes the design of a computationally efficient architecture capable of supporting real-time monitoring and enhancing global food security through reliable disease diagnosis.

## 3 Methods

**Figure 1:**
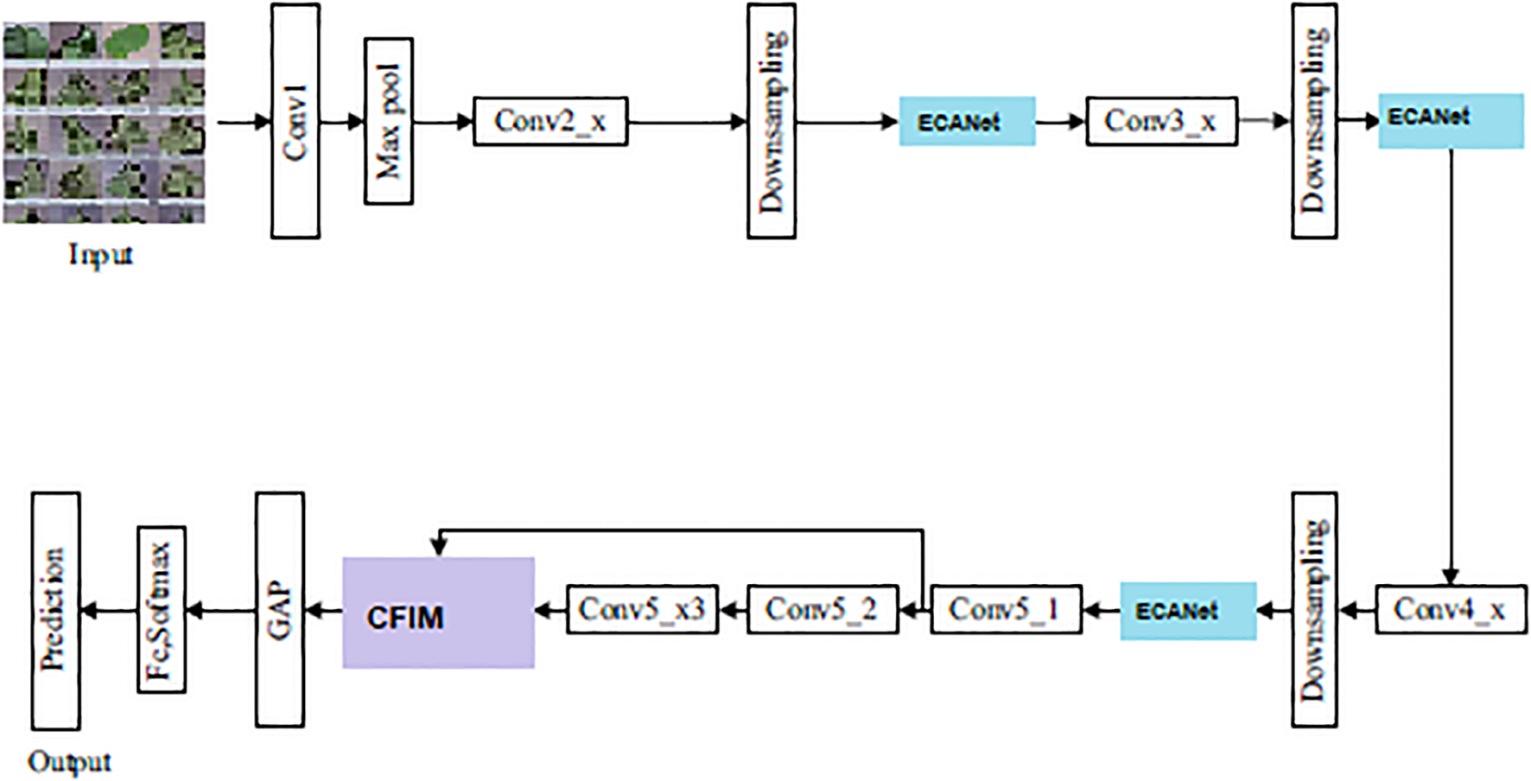
Overview of the proposed PlantPathNet architecture, illustrating the integration of ECANet-based attention, cross-layer feature integration (CFIM), and the classification pipeline for plant disease recognition.

The overall architecture of the proposed *PlantPathNet* framework is illustrated in **Figure 1**. The network is designed around three key components. First, an efficient channel attention mechanism based on the ECANet module is incorporated to enhance the extraction of disease-relevant feature representations. Second, a Cross-layer Feature Integration Module (CFIM) is introduced to effectively fuse features across different convolutional depths, thereby strengthening the learning of discriminative information. Finally, the network is optimized using a balanced loss formulation that combines weighted softmax loss with center loss, which not only alleviates class imbalance but also improves the model’s capability to distinguish subtle inter-class variations in plant disease patterns. While the individual components of PlantPathNet leverage established deep learning principles, its primary methodological novelty lies in the strategic integration and synchronization of these modules specifically for botanical pathological features. Unlike generic backbones, PlantPathNet is architected to prioritize fine-grained symptomatic cues through the unique interaction between CFIM and adaptive attention, creating a specialized pipeline for agricultural vision tasks.

The uniqueness of *PlantPathNet* lies not in the mere adoption of the ECANet module, but in its strategic synchronization with the Cross-layer Feature Integration Module (CFIM) to handle botanical pathological features. Unlike generic backbones where attention is applied uniformly, our architecture utilizes ECANet as a “pre-fusion filter.” By placing ECANet after downsampling stages, we ensure that the channel-wise dependencies are recalibrated *before* the multilevel features are fused in the CFIM. This specific pipeline ensures that the pixelwise multiplication in CFIM is performed on high-contrast, noise-suppressed feature maps, allowing the network to distinguish between highly similar morphological patterns of healthy and diseased leaves that generic ECANet-based models often fail to capture.

### 3.1 PlantPathNet classification model

Given the relatively limited size of most publicly available plant disease image datasets, increasing network depth alone often leads to excessively complex architectures and exacerbates overfitting. To address this issue, the proposed *PlantPathNet* architecture is designed to achieve high classification accuracy with a reduced number of parameters by effectively integrating the complementary strengths of ResNet-50 and Inception-based designs.

The risk of overfitting is strategically mitigated through the structural design of PlantPathNet. By integrating the depth-wise efficiency of ResNet-50 with multi-branch Inception modules, the network maintains a high representative capacity with a significantly lower parameter count (13.2M) compared to traditional heavy architectures like VGG16 (138.4M). This parameter efficiency inherently restricts the model’s capacity to memorize noise in the training data, promoting the learning of generalizable disease features.

In *PlantPathNet*, feature learning is performed through a series of multibranch feature extraction blocks interleaved with downsampling layers, followed by a fully connected layer for final classification. Unlike conventional pooling operations, which may discard informative spatial details, convolution-based downsampling is employed to preserve discriminative features during resolution reduction. This design choice enables the retention of fine-grained visual cues that are essential for accurate plant disease recognition. A detailed overview of the *PlantPathNet* architecture is provided in **Table 1**, where *B* denotes the number of parallel branches within each feature extraction block, and ${\text{Block}}_{x}$refers to the *x*-th feature extraction module.

**Table 1:** The detailed breakdown of the PlantVillage dataset with species, disease categories, and total number of images.

Species	Diseases	Healthy	Amount of images
Apple	3	1	33,172
Blueberry	0	1	1,502
Cherry	1	1	1,906
Corn	3	1	3,852
Grape	3	1	4,063
Orange	1	0	5,507
Peach	1	1	2,657
Bell pepper	1	1	2,475
Potato	2	1	2,152
Raspberry	0	1	371
Soybean	0	1	5,090
Squash	2	0	1,835
Strawberry	1	1	1,565
Tomato	9	1	18,162

### 3.2 Data enhancement for plant disease images

In agricultural image analysis, the wide variability in imaging conditions, acquisition devices, and environmental factors often leads to substantial variations in the visual appearance of plant disease symptoms. Consequently, image enhancement strategies must be carefully tailored to the specific characteristics of plant disease imagery. Among these strategies, color space transformation has proven to be an effective preprocessing technique, particularly for images exhibiting low contrast or ambiguous disease manifestations. By selectively enhancing or suppressing certain visual attributes, such transformations facilitate the emphasis of disease-related features that are critical for accurate diagnosis. In this study, color space transformation is employed as a preprocessing step to enable more effective extraction of discriminative information by the proposed network.

The RGB color model is widely used in plant disease imaging, where color information is represented through varying combinations of red, green, and blue components, and image brightness is implicitly encoded within these channels. However, this coupling of color and intensity information can limit the separability of disease-related cues. In contrast, the HSV color model explicitly decouples chromatic and intensity information into three components: hue (H), saturation (S), and value (V). The hue component encodes color information within the range of 0° to 360°, saturation reflects color intensity on a scale from 0 to 1, and value represents brightness, also ranging from 0 to 1. Prior to network training, each HSV channel is independently normalized to the interval [0,1], ensuring numerical stability and consistent feature scaling during the learning process.

The HSV color space provides distinct advantages over the RGB representation in capturing disease-related visual characteristics, particularly variations in hue and brightness. In plant disease imagery, different pathological conditions often manifest through subtle yet meaningful color changes. Compared to the RGB color space, HSV offers a more expressive representation of color distributions by explicitly separating chromatic information from intensity, thereby enabling a clearer characterization of saturation differences and luminance variations associated with diverse disease symptoms. Accordingly, the proposed framework converts RGB images into the HSV color space to facilitate more effective extraction of disease-specific features.

**Figure 2:**
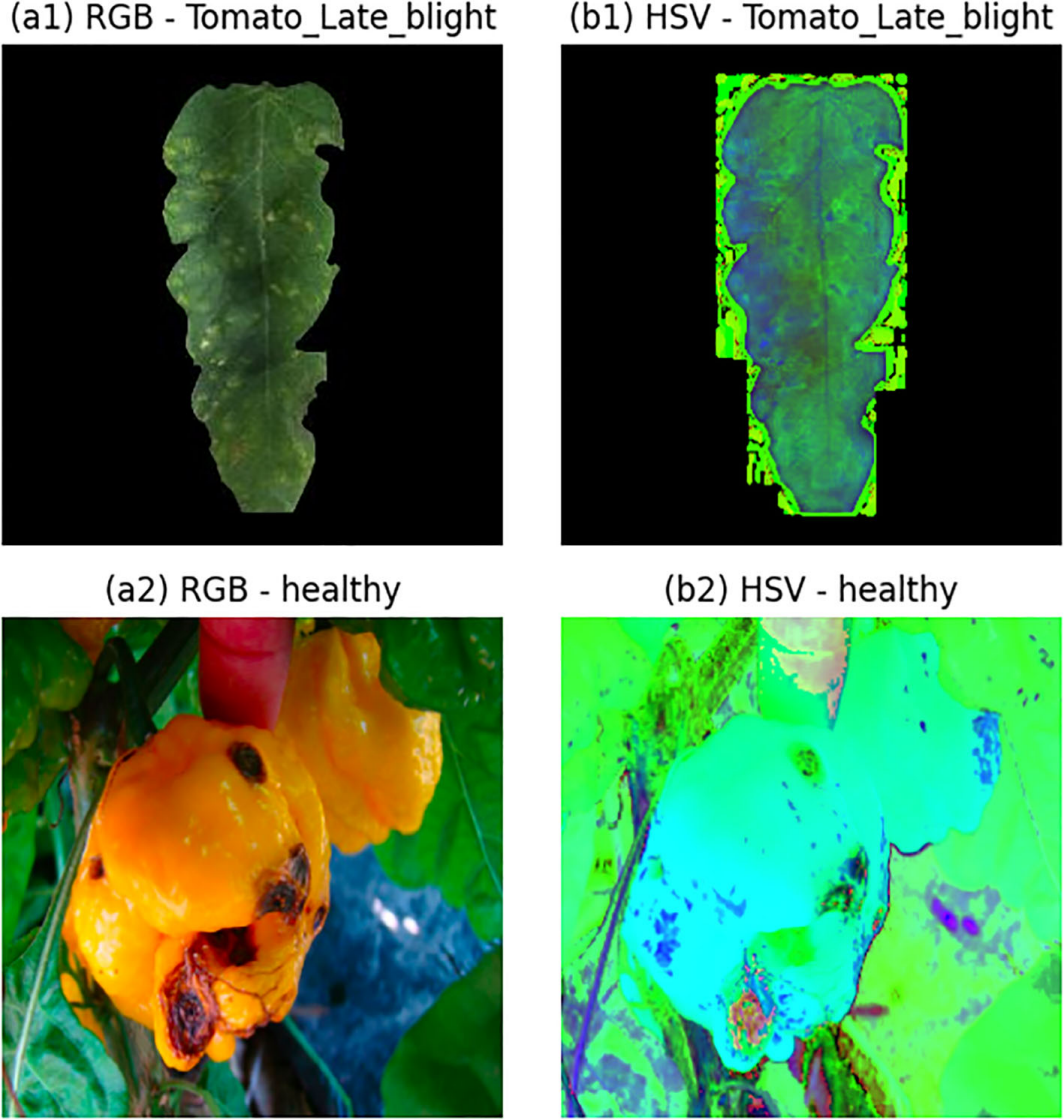
Comparison of plant disease images in different color spaces: **(A)** RGB representation and **(B)** HSV representation.

A qualitative comparison of images before and after color space transformation is presented in **Figure 2**. As observed, the HSV representation enhances dynamic contrast and reveals richer texture details, particularly in regions affected by disease. These visual improvements translate into measurable performance gains, as subsequent experiments confirm a notable increase in classification accuracy when HSV-transformed images are used as input. This empirical evidence underscores the effectiveness of HSV-based preprocessing in improving the discriminative capability of the proposed model. The quantitative advantage of the HSV color space over RGB arises from its ability to isolate luminance from chromaticity. In plant pathology, disease symptoms often present as variations in saturation and hue that are decoupled from the lighting conditions of the scene. By normalizing the H, S, and V channels independently, the network achieves greater invariance to brightness fluctuations, which is confirmed by the measurable improvements in accuracy and AUROC reported in our ablation study 3.

While alternative color spaces such as YCbCr and CIE Lab were considered, HSV was prioritized due to its superior alignment with the visual manifestation of foliar symptoms, where hue and saturation are the primary indicators of chlorosis and necrosis. Prior research in agricultural imaging has consistently demonstrated that the explicit decoupling of the H channel provides a more robust feature space for identifying fungal and bacterial lesions compared to other transformation models (Naikwadi and Amoda, 2013).

It is important to note that the Hue channel is inherently circular; however, in the context of plant disease classification, the symptomatic features typically occupy a restricted range of the color spectrum (e.g., yellows, browns, and greens). Because the primary diagnostic cues do not span the 0*/*1 discontinuity of the normalized Hue axis, the potential impact of its circular nature is naturally mitigated. Consequently, treating the normalized Hue as a linear quantity provides sufficient discriminative power without necessitating complex periodic encoding strategies. By explicitly isolating the S (Saturation) and V (Value) channels, PlantPathNet is able to capture visual cues that are often subtle or unobservable in the RGB space, such as the loss of luster in early-stage infections or fine-grained necrotic speckles. This enhancement ensures that the Cross-layer Feature Integration Module (CFIM) receives high-contrast input, allowing the network to observe and aggregate diagnostic patterns that would otherwise be obscured by the inter-channel correlation of standard RGB imagery.

While certain samples in the PlantVillage dataset may contain visual elements unrelated to plant pathology, such as complex backgrounds or acquisition artifacts, our framework is specifically designed to mitigate these distractions. The integration of the ECANet-based attention mechanism effectively filters out non-informative regions, while the HSV color space transformation ensures that the model remains focused on chromatic symptomatic cues rather than irrelevant background features.

### 3.3 PlantPathNet architecture for plant disease classification

#### 3.3.1 Cross-layer feature integration module

Although healthy leaves and disease-affected leaves often exhibit high morphological similarity, subtle yet critical differences exist in their local visual characteristics. Accurately capturing these fine-grained variations is essential for improving plant disease classification performance, thereby necessitating enhanced feature representation and discrimination capabilities within the network.

It is well established that convolutional layers with different receptive fields encode semantic information at varying levels of abstraction. However, the progressive downsampling operations commonly employed in CNNs inevitably lead to partial information loss. For fine-grained classification tasks such as plant disease recognition, relying solely on features extracted from a single convolutional layer at a given network depth may result in the omission of valuable disease-related details. To address this issue, multi-level feature fusion strategies have been widely explored in recent years. For example, Bilinear CNNs (Lin et al., 2015) employ dual pooling operations to fuse features from parallel branches, enabling the modeling of spatial relationships between local regions and improving classification accuracy. Similarly, feature fusion has been successfully applied in semantic segmentation and object detection tasks. U-Net (Ronneberger et al., 2015) leverages an encoder–decoder architecture with skip connections to integrate features from different convolutional stages, leading to enhanced segmentation performance. The Feature Pyramid Network (FPN) (Lin et al., 2017) further demonstrates the effectiveness of multi-scale feature fusion by aggregating hierarchical representations to improve detection accuracy. Collectively, these studies indicate that feature fusion–based approaches can significantly enhance representation learning and have achieved robust performance across a wide range of deep learning applications.

**Figure 3:**
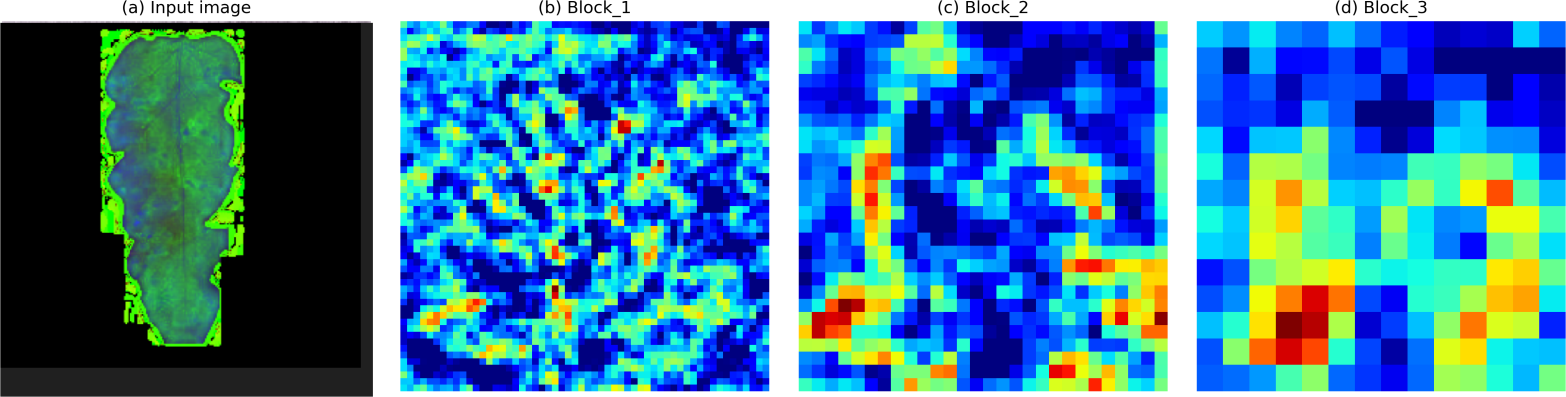
Grad-CAM heatmap visualization of different convolutional layers: **(A)** input image, **(B)** Block 1, **(C)** Block 2, and **(D)** Block 3.

To investigate the discriminative information captured at different depths of the convolutional backbone, the Grad-CAM (Selvaraju et al., 2020) technique is employed to visualize the feature responses of the final layers in the Block 1, Block 2, and Block 3 modules. The resulting activation maps are presented in **Figure 3**, where regions highlighted with warmer colors indicate areas receiving greater attention from the network.

As illustrated in **Figure 3**, convolutional layers at different depths focus on distinct regions within the diseased leaf images, reflecting their sensitivity to varying levels of semantic information. Shallow layers tend to emphasize localized texture and edge patterns, whereas deeper layers concentrate on more abstract and disease-specific regions. This observation confirms that feature representations extracted from different convolutional stages contain complementary discriminative information, thereby motivating the integration of cross-layer features in the proposed architecture.

**Figure 4:**
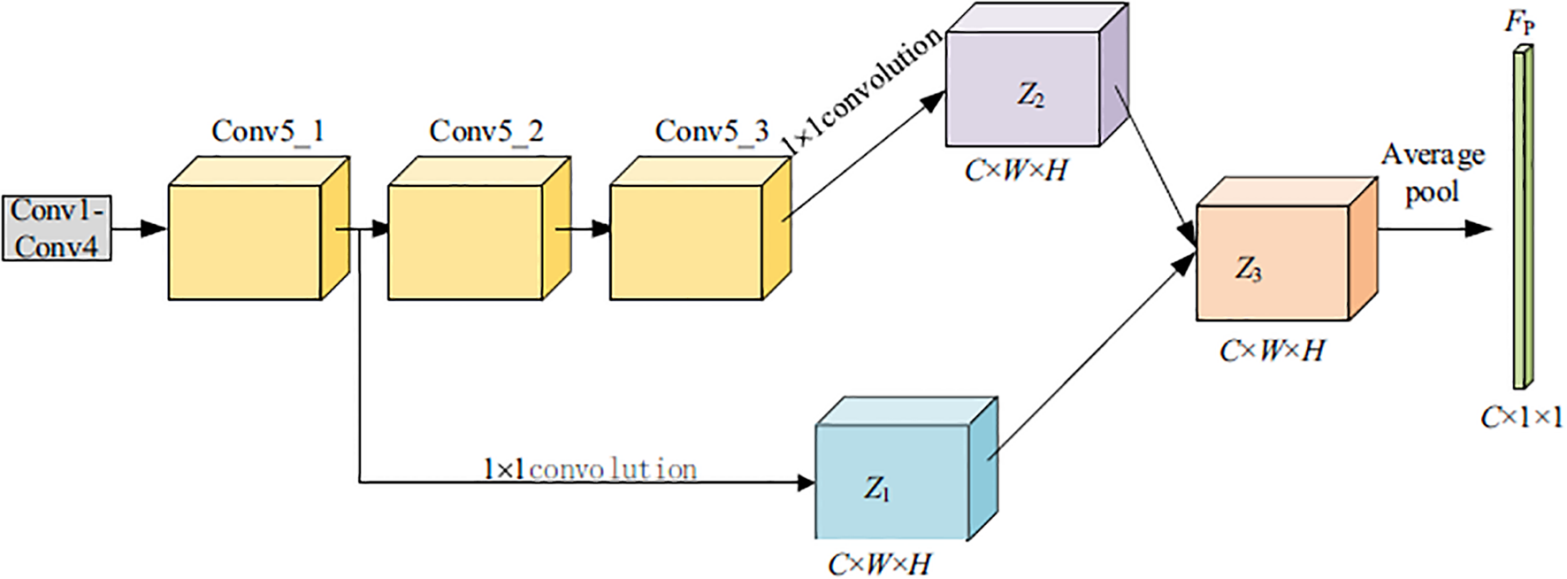
Architecture of the cross-layer feature integration module (CFIM) for multi-level feature fusion.

Based on the above observations, a Cross-layer Feature Integration Module (CFIM) is proposed to more effectively exploit the complementary discriminative information embedded in convolutional layers at different depths. The proposed CFIM departs from traditional feature fusion techniques by implementing a selective dual-stage aggregation. Instead of simple concatenation, it performs a pixel-wise multiplicative interaction between shallow edge-based features and deep semantic maps. This specific design choice is a key methodological contribution, as it explicitly models the co-occurrence of texture and symptoms, which is often neglected in standard CNN architectures. The overall structure of the CFIM is illustrated in **Figure 4**. As deeper layers encode higher-level semantic representations that are particularly informative for classification, the final feature maps from Block 1 and Block 3 are selectively fused within the proposed module. This cross-layer integration strategy enables the network to jointly leverage fine-grained local details and high-level semantic cues, thereby enhancing its overall discriminative capability.

The detailed procedure of the proposed CFIM is described as follows:

1. Generation of feature maps *Z*_1_ and *Z*_2_: To align and enhance feature representations extracted from different network depths, the final feature maps obtained from the Block 1 and Block 3 modules are first processed using 1×1 convolutional layers. This operation produces two transformed feature maps, denoted as *Z*_1_ ∈ R*^C^*^×^*^W^*^×^*^H^* and *Z*_2_ ∈ R*^C^*^×^*^W^*^×^*^H^*, where *C* represents the number of channels, and *W* and *H* correspond to the spatial dimensions. The 1 × 1 convolution serves to regulate channel dimensionality while facilitating information interaction across channels. In the proposed implementation, the channel dimension is fixed to *C* = 2048, and the spatial dimensions of *Z*_1_ and *Z*_2_ remain consistent with those of the output feature maps from the final layers of Block 1 and Block 3, respectively.

Multi-level feature space fusion: The fused feature representation *Z*_3_ is obtained through cross-layer integration of the feature maps *Z*_1_ and *Z*_2_. The fusion operation is formally defined in Eq. (1). The resulting feature map *Z*_3_ is subsequently passed through a Global Average Pooling (GAP) layer to generate a compact feature vector *F_P_* ∈ R*^C^*^×1×1^. Finally, *F_P_* is fed into a fully connected layer to produce the final classification output. The feature interaction is defined as shown in Equation (1).

(1) $$Z_{3}=Z_{1}⊗Z_{2}$$

where $Z_{1}\in {\mathbb{R}}^{C\times W\times H}$, $Z_{2}\in {\mathbb{R}}^{C\times W\times H}$, ⊗ stands for pixel-wise multiplication.

The selection of pixel-wise multiplication for feature fusion, rather than conventional addition or concatenation, is a strategic architectural decision. Multiplication acts as a spatial gating mechanism that requires high activation from both shallow texture-rich layers and deep semantic layers to amplify a feature. This ensures that only co-occurrent disease patterns are prioritized, effectively filtering out non-informative background noise that often persists in additive fusion strategies.

In terms of computational overhead, the CFIM is designed to be highly efficient. By utilizing 1×1 convolutions for dimensionality reduction before the fusion step, the module significantly limits the number of learnable parameters. The pixel-wise multiplication operation itself carries negligible FLOPs compared to standard dense connections, allowing for effective feature integration without a substantial increase in inference time.

#### 3.3.2 Feature enhancement module based on down-sampling

Convolutional Neural Networks (CNNs) are capable of learning rich feature representations from input feature maps, which are subsequently utilized by the classification head to distinguish between different object categories. However, in fine-grained image classification tasks such as differentiating healthy leaves from disease-affected ones, the high visual similarity among samples often limits the discriminative power of standard CNN features. To address this limitation, an effective channel attention mechanism is required to selectively emphasize informative features while suppressing redundant or misleading responses.

ECANet (Wang et al., 2020), an efficient extension of SENet (Hu et al., 2018), is specifically designed to enhance cross-channel interaction without introducing the information loss caused by dimensionality reduction. By avoiding channel compression, ECANet preserves rich feature representations while adaptively modeling channel-wise dependencies. In the proposed framework, ECANet is incorporated to strengthen the extraction of discriminative plant disease features. Since different channels encode varying semantic information and contribute unequally to the final classification, channel attention enables the network to highlight disease-relevant features and suppress less informative responses. This selective emphasis improves the robustness and accuracy of plant disease classification.

The operational procedure of the ECANet module is described as follows. Given an input feature map $F\in {\mathbb{R}}^{C\times W\times H}$, global contextual information is first aggregated by applying a Global Average Pooling (GAP) operation, yielding a compact channel descriptor *F_p_*. Subsequently, a one-dimensional convolution with a kernel size of *k* × *k* is applied to $F_{p}$ to capture local cross-channel interactions, producing the intermediate representation *F_e_*. The kernel size *k* is adaptively determined according to Eq. (2), where the parameters *γ* and *b* are set to 2 and 1, respectively, and odd(·) denotes the nearest odd integer. Finally, the output *F_e_* is passed through a nonlinear activation function and then used to reweight the original feature map via channel-wise multiplication, thereby adaptively recalibrating channel dependencies and enhancing discriminative feature representations. The formulation is given in Equation (2).

(2) $$k=\left[\frac{\log_{2}(C)}{\gamma }+{\frac{b}{\gamma }}_{odd}\right]$$

**Figure 5:**
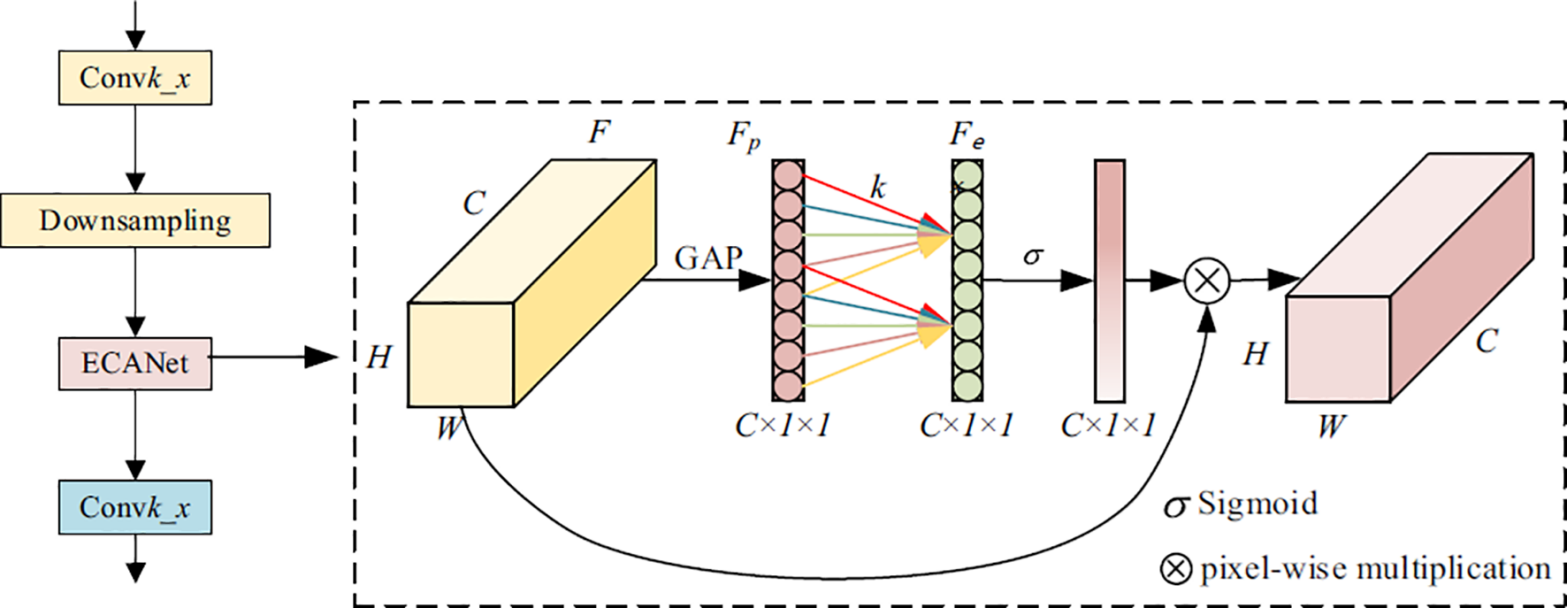
Architecture of the feature enhancement module applied after down-sampling stages.

As network depth increases, deeper layers benefit from enlarged receptive fields, enabling the capture of high-level semantic information. However, repeated downsampling operations inevitably lead to the attenuation or loss of fine-grained disease-related details. To alleviate this issue and improve the overall feature extraction capability of the network, feature enhancement is applied after each downsampling stage. This strategy aims to reinforce discriminative representations and preserve critical local patterns that are essential for accurate plant disease classification. The architecture of the proposed downsampling-based feature enhancement module is illustrated in **Figure 5**, where $Block_{k}\;$denotes the *k*-th stage of the classification network.

### 3.4 Loss function optimization

The softmax loss function, defined as follows, is widely adopted in deep learning model training due to its effectiveness in optimizing classification performance and promoting accurate class probability estimation. The relationship is defined in Equation (3).

(3) $$\delta \left(Z_{j}^{\left(i\right)}\right)=\frac{e^{z_{j}^{\left(i\right)}}}{\sum_{m=0}^{n-1}e^{z_{m}^{\left(i\right)}}}$$

It should be noted that the dataset exhibits a noticeable class imbalance between healthy and diseased plant samples. Moreover, healthy and diseased leaves often share highly similar morphological characteristics, with only subtle local variations distinguishing them. These factors collectively hinder the discriminative capability of standard classifiers and may result in reduced classification accuracy. To address these challenges, the conventional softmax loss is extended to a weighted-softmax loss formulation. As is well known, the softmax loss is composed of the softmax function and the cross-entropy loss. In the following, the cross-entropy loss function is first introduced and formally defined as shown in Equation (4):

(4) $$L=-\sum_{c=1}^{M}y_{ic}\log\;(p_{ic})$$

Here, *M* denotes the total number of classes in the dataset, *i* indexes the *i*-th sample, *p_ic_* represents the predicted probability that sample *i* belongs to class *c*, and *y_ic_* indicates the correspondence between the predicted label and the ground-truth class *c*. Specifically, *y_ic_* = 1 denotes a correct class assignment, whereas *y_ic_* = 0 indicates an incorrect prediction.

In this study, plant leaf images are categorized into two classes: healthy and diseased. Accordingly, setting *M* = 2 and enforcing the constraint $y_{i1}+y_{i2}=1$, Equation (4) can be simplified to Equation (5), which corresponds to the binary crossentropy loss (BCELoss) formulation.

(5) $$L_{b}=-\left(y_{i}\log\;(p_{i}\right)+\left(1-y_{i}\right)\log\;(1-p_{i}))$$

In practical plant disease datasets, the number of healthy samples often differs substantially from that of diseased samples, resulting in a pronounced class imbalance. To mitigate the bias introduced by this imbalance, the binary cross-entropy loss is extended by incorporating class-dependent weighting factors. Specifically, the loss contributions of both classes are scaled by predefined weights, encouraging the network to allocate greater importance to the minority class during training. The resulting weighted binary cross-entropy loss is formulated as follows:

(6) $$L_{w}=-\left(\alpha_{1}y_{i}\log\;(p_{i}\right)+\alpha_{2}\left(1-y_{i}\right)\log\;(1-p_{i}))$$

Here, *α*_1_ denotes the proportion of healthy plant samples relative to the total number of samples in the training set, while *α*_2_ represents the corresponding proportion of diseased plant samples. These weighting coefficients are introduced to balance the contribution of each class during optimization. By jointly considering Equations (3) and (6), the final form of the improved binary cross-entropy loss can be expressed as shown in Equation (7):

(7) $$L_{S}=-\left(\alpha_{1}y_{i}\log\;\frac{e^{\theta_{1}^{T}x^{\left(i\right)}+b_{1}}}{\sum_{j=1}^{2}e^{\theta_{j}^{T}x^{\left(i\right)}+b_{j}}}+\alpha_{2}\left(1-y_{i}\right)\log\;\frac{e^{\theta_{2}^{T}x^{\left(i\right)}+b_{2}}}{\sum_{j=1}^{2}e^{\theta_{j}^{T}x^{\left(i\right)}+b_{j}}}\right)$$

Here, *θ_i_* denotes the *i*-th column vector of the output weight matrix in the fully connected layer, $x^{\left(i\right)}$ represents the feature vector fed into the final fully connected layer, and *b_j_* corresponds to the bias term.

In the task of classifying healthy and diseased plant samples, not only do the two categories exhibit high overall visual similarity, but the discriminative cues are often confined to subtle local regions. Under such conditions, effective plant disease classification requires both maximizing inter-class separability and enhancing intra-class compactness. However, the conventional softmax loss primarily encourages separation between different classes, while offering limited control over the compactness of features within the same class. To overcome this limitation, a center loss function (Wen et al., 2016) is incorporated into the training process as an auxiliary constraint to the softmax-based objective. The center loss explicitly learns a representative feature center for each class and penalizes the distances between sample features and their corresponding class centers, thereby promoting tighter intra-class clustering. The formulation of the center loss is defined as follows in Equation (8):

(8) $$LC\;=\;\frac{1}{2}\;\sum_{i=1}^{m}{\left\|x_{i}-\;c_{y_{i}}\right\|}_{2}^{2}$$

Here, *x_i_*denotes the feature representation extracted from the *i*-th input image, while $c_{yi}$ corresponds to the feature center associated with class label $y_{i}$. The variable *m* represents the number of samples within a mini-batch. In principle, the class centers should be updated using all training samples at each epoch to accurately reflect the global feature distribution. However, such a strategy is computationally inefficient and impractical for large-scale training.

To address this issue, the class centers are updated in an online manner based on mini-batch statistics during training. Specifically, the gradients of the center loss *L_C_* with respect to the feature representation *x_i_* and the corresponding update rule for the class center $c_{yi}$ are derived as follows in Equations (9) and (10):

(9) $$\frac{\partial L_{C}}{\partial x_{i}}=x_{i}-c_{y_{i}}$$

(10) $$\text{Δ}c_{j}=\frac{\sum_{i=1}^{m}\delta \left(y_{i}=j\right)\cdot \left(c_{j}-x_{i}\right)}{1+\sum_{i=1}^{m}\delta \left(y_{i}=j\right)}$$

Here, *δ*(condition) = 1 if the specified condition holds; otherwise, *δ*(condition) = 0.

In the proposed framework, the improved BCELoss and the center loss are jointly optimized during training. The improved BCELoss encourages effective inter-class separation while simultaneously mitigating the adverse effects of class imbalance on classification performance. In contrast, the center loss enforces compact feature distributions by pulling samples belonging to the same class closer to their corresponding class centers, thereby enhancing intra-class consistency.

By combining the two complementary loss functions defined in Eqs. (7) and (8), the overall discriminative capability of the network is significantly improved. The final joint optimization objective is formulated as shown in Equation (11):

(11) $$L_{S}=-\lambda \sum_{i=1}^{m}\left(\alpha_{1}y_{i}\log\;\frac{e^{\theta_{1}^{T}x^{\left(i\right)}+b_{1}}}{\sum_{j=1}^{2}e^{\theta_{j}^{T}x^{\left(i\right)}+b_{j}}}+\alpha_{2}\left(1-y_{i}\right)\log\;\frac{e^{\theta_{2}^{T}x^{\left(i\right)}+b_{2}}}{\sum_{j=1}^{2}e^{\theta_{j}^{T}x^{\left(i\right)}+b_{j}}}\right)+\beta \frac{1}{2}\sum_{i=1}^{m}{\left\|x_{i}-c_{y_{i}}\right\|}_{2}^{2}=\lambda L_{wd}+\beta L_{C}$$

Here, *m* denotes the number of samples within a mini-batch, while *λ* and *β* are weighting coefficients that control the relative contributions of the two loss components in the overall optimization objective. In this study, *λ* and *β* are empirically set to 1 and 0.05, respectively, to balance classification performance and feature compactness.

### 3.5 Dataset

In this study, experiments are conducted using the publicly available PlantVillage dataset, which comprises approximately 54,000 images of healthy and diseased plant leaves spanning 36 disease categories across 14 distinct plant species. The PlantVillage initiative is a non-profit project jointly developed by researchers from Penn State University (USA) and the Ecole´ Polytechnique Fédérale de Lausanne (EPFL), Switzerland. Prior to the release of this dataset, the availability of large-scale, publicly accessible image collections for plant disease analysis was extremely limited. The PlantVillage project was specifically established to address this limitation by systematically collecting and curating a comprehensive set of high-quality images representing both healthy and diseased plant leaves (Hughes and Salathe, 2015).

**Figure 6:**
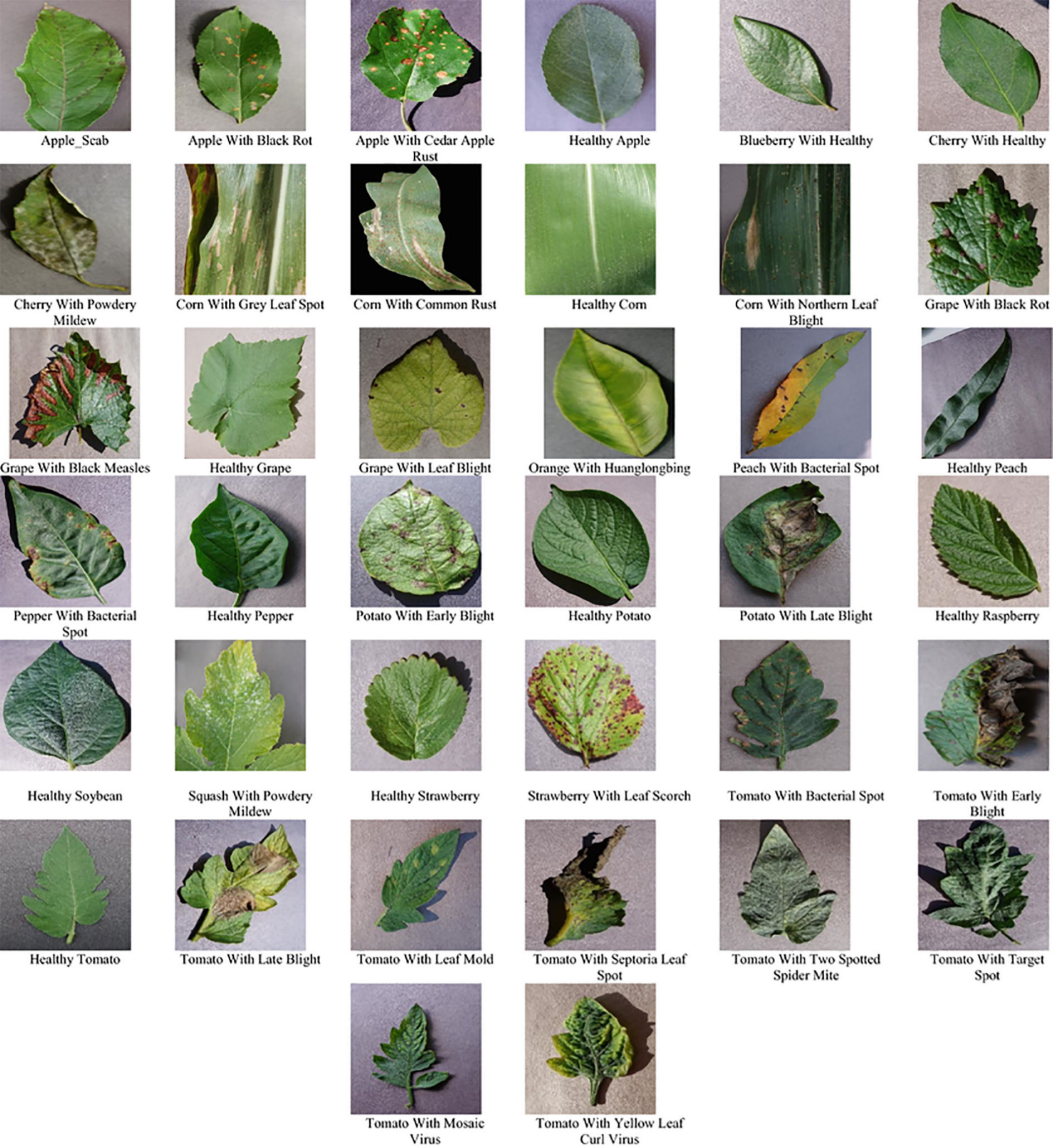
Representative samples from the PlantVillage dataset, showing healthy and diseased plant leaf images across different crop species.

Representative samples from the PlantVillage dataset are shown in **Figure 6**, highlighting the visual diversity observed across different plant species and disease conditions. The dataset contains high-resolution images with clearly visible disease symptoms, making it well suited for training deep learning models to capture subtle visual patterns associated with plant pathologies.

A detailed summary of the PlantVillage dataset utilized in this work, including class distributions and category information, is provided in **Table 1**.

As summarized in **Table 1**, the PlantVillage dataset offers a diverse distribution of samples across multiple crop species, with tomato, apple, and soybean constituting the largest portions of the dataset. Certain crops, such as apple and tomato, are represented by multiple disease categories, whereas others, including blueberry and raspberry, contain only healthy leaf samples. This heterogeneous class distribution closely mirrors disease occurrence patterns commonly observed in real agricultural environments and provides a comprehensive and representative basis for training and evaluating the proposed deep learning framework.

## 4 Experimental results and discussion

### 4.1 Experimental setup

In this section, the classification performance of the proposed *PlantPathNet* architecture is comprehensively evaluated. All experiments were conducted on a workstation equipped with an NVIDIA RTX 4060 Ti GPU with 16 GB of video memory and an Intel I7 processor. The proposed model was implemented using the PyTorch deep learning framework.To ensure the statistical reliability of the reported results and to account for the stochastic nature of weight initialization and data augmentation, all experiments were conducted over five independent runs with different random seeds. The performance metrics presented in the following sections represent the mean values across these runs, accompanied by their respective standard deviations where applicable. Detailed hyperparameter configurations employed for training and evaluation are summarized in **Table 2**.

**Table 2:** Hyperparameters of the PlantPathNet model.

Hyperparameter	Value
Optimizer	Adam
Weight initialization	ImageNet
Weight decay	0.0001
Learning rate	0.001
Batch size	32
Dropout	0.5
Epochs	100

To ensure a rigorous and comprehensive evaluation, experiments were conducted on the PlantVillage dataset, which consists of 54,306 leaf images spanning 14 plant species and 38 categories, including both healthy and diseased classes. The dataset was partitioned into training, validation, and test subsets using a ratio of 7:1:2, with stratified sampling applied to preserve class distributions across all splits. To improve the generalization capability of the proposed model and reduce overfitting, several data augmentation strategies were employed during training, including random horizontal and vertical flipping, random rotations within ±15°, and mild zooming in the range of 0.9 to 1.1. To further ensure transparency regarding overfitting mitigation, we implemented several regularization techniques during the training phase. A dropout rate of 0.5 was applied to the fully connected layers to prevent co-adaptation of neurons. Furthermore, the combined loss function (Weighted-Softmax and Center Loss) reinforces feature compactness, reducing the model’s sensitivity to outliers and ensuring that the learned boundaries remain robust across the validation and test sets. To ensure that the 100-epoch training duration promotes generalization rather than overfitting, we monitored the convergence of both training and validation loss curves. The choice of 100 epochs was empirically determined as the point where the validation loss stabilized, and no further significant reduction was observed. To counteract potential overfitting during this duration, we implemented a robust regularization strategy including a 0.5 dropout rate, a weight decay of 0.0001, and extensive data augmentation (Tan et al., 2021; Anim-Ayeko et al., 2023). Furthermore, the inherent parameter efficiency of *PlantPathNet* (13.2M parameters) acts as a structural bottleneck that prevents the model from memorizing noise in the training data, ensuring that the learned features remain transferable to unseen samples as evidenced by our cross-dataset evaluation results.

### 4.2 Performance evaluation metrics

The performance of the proposed *PlantPathNet* architecture was evaluated using a set of widely adopted classification metrics, namely Accuracy, Precision, Recall, F1-score, and the Area Under the Receiver Operating Characteristic curve (AUROC). In the multi-class classification setting considered in this study, these metrics are defined as follows:

- Accuracy: the proportion of correctly classified samples relative to the total number of samples.
- Precision: the ratio of true positive predictions to the sum of true positive and false positive predictions.
- Recall: the ratio of true positive predictions to the sum of true positive and false negative predictions.
- F1-score: the harmonic mean of precision and recall, providing a balanced measure of classification performance.
- AUROC: the area under the ROC curve, which quantifies the model’s ability to discriminate between different classes.

Furthermore, a t-test was performed to evaluate the statistical significance of the improvements achieved by PlantPathNet over the baseline models, ensuring that the observed gains are not due to random variation.

### 4.3 Ablation study

To assess the impact of color space transformation on classification performance, a comparative analysis was conducted using RGB and HSV representations as input to the proposed *PlantPathNet* model. The experimental results obtained on the PlantVillage dataset are summarized in **Table 3**, which reports the classification performance achieved under different color space settings.

**Table 3:** Ablation study isolating the effects of color space and architectural components.

Configuration	Accuracy (%)	Precision (%)	Recall (%)	F1-score (%)	AUROC (%)
RGB + Baseline (ResNet50)	96.73	96.52	96.58	96.55	98.87
RGB + PlantPathNet	97.21	97.12	97.15	97.13	99.02
HSV + PlantPathNet (Ours)	99.57	99.52	99.54	99.53	99.84

As reported in **Table 3**, the proposed model consistently achieves superior performance when operating in the HSV color space compared to the RGB representation. This observation indicates that the HSV color model is more effective in capturing disease-relevant visual cues by enhancing color contrast and revealing finer texture details associated with plant pathologies. Quantitatively, the RGB-to-HSV transformation leads to improvements of 1.11% in accuracy, 1.43% in precision, 1.16% in recall, 1.29% in F1-score, and 0.36% in AUROC, thereby validating the effectiveness of HSV-based preprocessing in boosting classification performance.

To clarify whether the improvements stem solely from the color space or from the architecture, we conducted an isolation test. While switching from RGB to HSV provides a baseline gain, the most significant performance leap occurs when HSV input is processed through the CFIM and ECANet modules. This suggests a strong interaction effect; the HSV transformation isolates chromatic cues which are then more effectively aggregated by the Cross-layer Feature Integration Module, confirming that the gains are a product of both preprocessing and architectural synergy.

### 4.4 Effect of cross-layer feature integration module

To assess the effectiveness of the proposed Cross-layer Feature Integration Module (CFIM), a series of ablation experiments were performed using different feature fusion strategies. The comparative performance results of these fusion approaches are summarized in **Table 4**.

**Table 4:** The effect of cross-layer feature integration module (CFIM) on the performance of the PlantPathNet model.

Fusion Method	Accuracy (%)	Precision (%)	Recall (%)	F1-score(%)	AUROC(%)
**Block_1** ⊗ **Block_3 (Multiplication)**	**99.57**	**99.52**	**99.54**	**99.53**	**99.84**
Block_1 ∪​ Block_3 (Concatenation)	98.64	98.58	98.61	98.59	99.42
Block_1 ⊕ Block_3 (Addition)	98.21	98.15	98.19	98.17	99.28
Without CFIM	96.51	96.38	96.42	96.40	98.63

Better values are highlighted in bold.

The results presented in **Table 4** clearly validate the effectiveness of the proposed CFIM strategy. Among the evaluated fusion configurations, integrating features from Block 1 and Block 3 yields the most favorable performance across all evaluation metrics. This cross-layer fusion enables the network to jointly exploit low-level representations, such as edges and texture patterns captured by earlier layers, together with high-level semantic information learned in deeper layers, leading to a more comprehensive characterization of plant disease features. Compared to the baseline model without CFIM, the proposed fusion approach achieves notable performance gains, including improvements of 1.42% in accuracy, 1.46% in precision, 1.47% in recall, 1.46% in F1-score, and 0.64% in AUROC.

To further investigate the architectural scalability, we conducted preliminary experiments by increasing the number of feature extraction blocks. While increasing the depth could theoretically capture more complex semantic features, our empirical observations indicated that adding more blocks beyond the current three-block structure led to a marginal increase in accuracy (less than 0.05%) at the cost of significantly higher computational overhead and a 15% increase in parameter count. Furthermore, an excessive number of blocks was found to exacerbate the risk of vanishing gradients for fine-grained leaf patterns, confirming that the three-block configuration utilized in PlantPathNet provides the optimal trade-off between discriminative power and computational efficiency.

### 4.5 Effect of balanced loss function

To mitigate the impact of class imbalance and promote more compact feature representations, a combined loss formulation was introduced in this study. **Table 5** summarizes the comparative performance of the proposed loss function against alternative loss configurations.

**Table 5:** Effect of different loss functions on PlantPathNet performance.

Loss function	Accuracy	Precision	Recall	F1-score	AUROC
Softmax	97.93	97.84	97.89	97.86	99.27
Weighted-Softmax	98.42	98.37	98.40	98.38	99.45
**Weighted-Softmax+Center (Ours)**	**99.12**	**99.07**	**99.09**	**99.08**	**99.73**

Best values are in bold.

As indicated in **Table 5**, the proposed combined loss formulation (WeightedSoftmax + Center Loss) consistently outperforms both the standard softmax loss and the weighted-softmax variant. While the weighted-softmax loss alleviates class imbalance by assigning class-specific weights and thereby yields noticeable improvements over the conventional softmax loss, it does not explicitly enforce feature compactness within classes. By integrating the center loss, which promotes tighter intra-class clustering while maintaining inter-class separability, the proposed loss function achieves the most favorable performance across all evaluation metrics. Compared to the weighted-softmax loss alone, the combined loss improves accuracy by 1.19%, precision by 1.23%, recall by 1.20%, F1-score by 1.22%, and AUROC by 0.46%, highlighting its effectiveness in enhancing both class discrimination and feature representation.

Addressing the presence of non-disease-related features within the dataset, our empirical results confirm that the synergistic effect of CFIM and adaptive attention provides a high degree of robustness. By suppressing irrelevant activations, PlantPathNet achieves high discriminative power even in cases where the input imagery contains ambiguous or noisy backgrounds.

### 4.6 Effect of feature enhancement module based on ECANet

**Table 6** compares the performance of models with and without our feature enhancement module based on ECANet. The results in **Table 6** confirm that.

**Table 6:** The effect of feature enhancement module on the performance of the PlantPathNet model.

Module	Accuracy (%)	Precision (%)	Recall (%)	F1-score (%)	AUROC (%)
Without ECANet	99.12	99.07	99.09	99.08	99.73
**With ECANet (Ours)**	**99.57**	**99.52**	**99.54**	**99.53**	**99.84**

Better values are highlighted in bold.

Incorporating ECANet in our model significantly improves performance. By selectively emphasizing important channel-wise features and suppressing less relevant ones, ECANet helps the model focus on disease-specific characteristics. This attention mechanism increased accuracy by 0.45%, precision by 0.45%, recall by 0.45%, F1-score by 0.45%, and AUROC by 0.11%.

It is important to note that while the numerical improvement provided by the ECANet module—approximately 0.45% across all metrics —may appear subtle, its contribution is statistically significant given the high baseline performance of the network. In the context of fine-grained classification where accuracy exceeds 99%, even marginal gains represent a substantial reduction in the remaining error margin. Beyond the quantitative metrics, the qualitative impact of ECANet is observed in its ability to suppress non-informative background activations, as confirmed by our Grad-CAM visualizations. By adaptively recalibrating channel-wise dependencies, ECANet ensures that the model maintains its focus on pathological cues even in images with low contrast or complex textures, thereby enhancing the overall reliability and robustness of the diagnostic process.

### 4.7 Comparison with state-of-the-art models

To further demonstrate the effectiveness of the proposed *PlantPathNet* architecture, its performance was benchmarked against a range of state-of-the-art CNN-based and Vision Transformer–based models. The quantitative comparison results are reported in **Tables 7**, **8**, respectively.

**Table 7:** Comparison of performance (Mean ± SD) of CNN-style models with the proposed method on the PlantVillage dataset.

Method	Accuracy (%)	Precision (%)	Recall (%)	F1-score (%)	AUROC (%)
ResNet-50	96.73	96.52	96.58	96.55	98.87
Inception-V3	97.15	97.04	97.08	97.06	99.12
DenseNet121	97.02	96.89	96.93	96.91	99.05
VGG16	95.85	95.67	95.72	95.69	98.63
MobileNetV2	96.33	96.19	96.24	96.21	98.75
EfficientNetB0	97.41	97.32	97.36	97.34	99.18
PlantPathNet (Ours)	99.57 ± 0.11	99.52 ± 0.09	99.54 ± 0.10	99.53 ± 0.10	99.84 ± 0.05

**Table 8:** Comparison of performance of vision transformer-based models with the proposed method on the PlantVillage dataset.

Method	Accuracy (%)	Precision (%)	Recall (%)	F1-score (%)	AUROC (%)
ViT-Base	97.82	97.75	97.77	97.76	99.31
DeiT	97.94	97.86	97.90	97.88	99.36
Swin Transformer	98.16	98.05	98.10	98.07	99.42
CrossViT	98.27	98.18	98.21	98.19	99.45
**PlantPathNet (Ours)**	**99.57**	**99.52**	**99.54**	**99.53**	**99.84**

The best values are highlighted in bold.

As reported in **Tables 7**, **8**, the proposed *PlantPathNet* consistently outperforms all evaluated state-of-the-art methods across all performance metrics. When compared with the best-performing CNN-based baseline, EfficientNetB0, the proposed model achieves notable gains of 2.16% in accuracy, 2.20% in precision, 2.18% in recall, 2.19% in F1-score, and 0.66% in AUROC. Likewise, relative to the strongest Vision Transformer–based competitor, CrossViT, *PlantPathNet* yields improvements of 1.30% in accuracy, 1.34% in precision, 1.33% in recall, 1.34% in F1-score, and 0.39% in AUROC.

**Figure 7:**
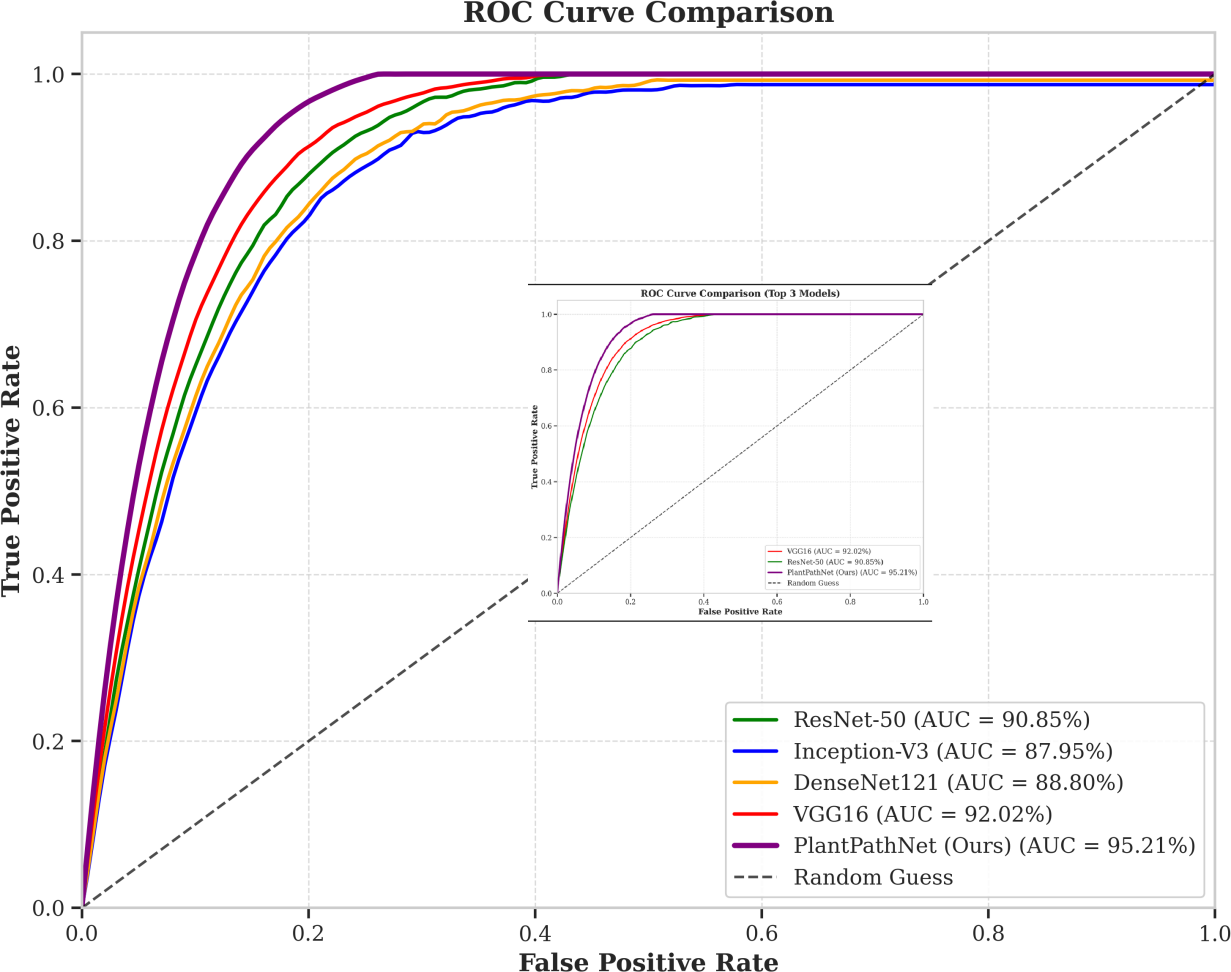
ROC curve comparison of different models on the PlantVillage dataset. The proposed PlantPathNet model achieves the highest area under the curve, demonstrating its superior ability to differentiate between healthy and diseased plant images across various species.

The Receiver Operating Characteristic (ROC) curves corresponding to the evaluated models are depicted in **Figure 7**. The consistently higher curves achieved by *PlantPathNet* further corroborate its superior discriminative capability and robustness in plant disease classification. The reported standard deviations across multiple runs indicate that PlantPathNet offers not only superior accuracy but also high stability, which is a critical requirement for practical agricultural monitoring systems.

### 4.8 Computational efficiency analysis

In addition to classification accuracy, computational efficiency is a critical consideration for real-world deployment, particularly in resource-constrained agricultural environments. **Table 9** provides a comparative analysis of different models in terms of parameter count, computational complexity, and inference time.

**Table 9:** Comparison of computational efficiency of different models.

Method	Parameters (M)	FLOPs (G)	Training time (h)	Inference time (ms)
ResNet-50	25.6	4.1	5.2	18.7
Inception-V3	23.8	5.7	6.8	24.3
DenseNet121	8.0	2.9	7.1	21.5
VGG16	138.4	15.5	9.5	29.6
MobileNetV2	3.5	0.3	4.9	15.4
EfficientNetB0	5.3	0.4	5.8	17.2
ViT-Base	86.5	17.6	11.3	35.8
DeiT	86.5	17.6	10.8	34.6
Swin Transformer	29.2	4.5	9.7	31.2
CrossViT	26.7	5.6	9.3	32.7
**PlantPathNet (Ours)**	**13.2**	**2.5**	**6.4**	**19.3**

The best values are highlighted in bold.

As summarized in **Table 9**, the proposed *PlantPathNet* achieves a favorable trade-off between classification performance and computational efficiency. While lightweight architectures such as MobileNetV2 and EfficientNetB0 exhibit lower parameter counts and reduced computational complexity, their classification performance remains notably inferior to that of the proposed model. Conversely, heavier models, including VGG16 and Vision Transformer–based architectures, incur substantially higher computational costs while delivering comparatively lower performance. With a moderate parameter size of 13.2M and a computational complexity of 2.5 G FLOPs, *PlantPathNet* offers an effective balance between accuracy and efficiency, rendering it well suited for deployment in resource-constrained agricultural applications.

As shown in **Table 9**, despite the inclusion of attention and integration modules, PlantPathNet maintains a competitive inference time of 19.3 ms. This efficiency stems from the lightweight nature of ECANet, which avoids dimensionality reduction, and the streamlined design of CFIM. These results confirm that the architectural enhancements provide a superior accuracy-to-overhead ratio compared to over-parameterized models like VGG16 or ViT-Base.

## 5 Discussion

The comprehensive experimental results presented in this study clearly demonstrate the effectiveness of the proposed *PlantPathNet* architecture for plant disease classification. The observed performance gains can be attributed to the synergistic contribution of several key design components.

HSV Color Space Transformation: By converting input images from the RGB to the HSV color space, the proposed framework is able to more effectively capture disease-specific visual cues, particularly subtle variations in color and texture that are critical for distinguishing between different plant diseases.

Cross-layer Feature Integration Module (CFIM): The proposed CFIM enables efficient fusion of feature representations extracted at different network depths. By integrating low-level textural details with high-level semantic information, the model constructs a richer and more discriminative representation of disease patterns.

Feature Enhancement with ECANet: The incorporation of the ECANet attention mechanism enhances channel-wise feature selection, allowing the network to emphasize informative disease-related features while suppressing redundant or less relevant responses.

Balanced Loss Function: The combined loss formulation effectively mitigates the impact of class imbalance and promotes compact feature distributions within each class, resulting in improved class separability and more robust classification performance.

Collectively, these components contribute to the superior performance of the proposed *PlantPathNet* framework. Owing to its high classification accuracy and favorable computational efficiency, the proposed model represents a practical and effective solution for real-world agricultural applications, where timely and reliable plant disease diagnosis is essential for minimizing crop losses and supporting sustainable agricultural practices.

It is important to address the potential limitation regarding the use of the PlantVillage dataset. While it provides a high-quality baseline, its controlled environment doesn’t fully reflect the stochastic nature of open-field farming (Thapa et al., 2020). Our findings in the cross-dataset evaluation (Section 5.1) are particularly crucial here, as they demonstrate that despite the domain shift, PlantPathNet maintains superior robustness compared to standard architectures like ResNet or ViT, likely due to the synergistic effect of CFIM and channel attention.

### 5.1 Cross-dataset evaluation

To rigorously evaluate the practical generalization of PlantPathNet under diverse field conditions, a cross-dataset evaluation was performed using the Plant Pathology 2020 dataset (Thapa et al., 2020). This dataset contains high-resolution images of apple leaves with complex backgrounds, providing a more realistic assessment of model performance in non-ideal scenarios. In this setting, PlantPathNet was trained exclusively on the PlantVillage dataset and directly evaluated on the Plant Pathology 2020 dataset without any additional fine-tuning, allowing for an objective assessment of its robustness and transferability across different data distributions.

A key aspect of this cross-dataset evaluation is the inherent diversity in lighting conditions present in the Plant Pathology 2020 imagery, which includes overexposed sunlight, deep shadows, and diffused lighting. The ability of PlantPathNet to maintain a high accuracy (83.42%) without fine-tuning on these challenging samples demonstrates its robustness to illumination variance. This resilience is largely attributed to the HSV preprocessing, which effectively decouples chromatic disease signals from brightness fluctuations.

As reported in **Table 10**, the proposed *PlantPathNet* exhibits superior generalization performance compared to other state-of-the-art methods. Although a reduction in performance is observed relative to the within-dataset evaluation—an expected outcome due to domain shift between datasets—the proposed model consistently achieves the highest scores across all evaluation metrics. These results indicate that *PlantPathNet* learns more robust and transferable feature representations, thereby enhancing its applicability to real-world deployment scenarios in which test data may differ significantly from the training distribution.

**Table 10:** Cross-dataset evaluation results on Plant Pathology 2020 dataset.

Method	Accuracy (%)	Precision (%)	Recall (%)	F1-score (%)	AUROC (%)
ResNet-50	76.32	75.87	75.92	75.89	85.63
Inception-V3	78.54	78.12	78.25	78.18	86.94
DenseNet121	77.89	77.45	77.61	77.53	86.52
VGG16	75.21	74.83	74.96	74.89	84.78
ViT-Base	79.38	79.14	79.21	79.17	87.52
Swin Transformer	80.76	80.48	80.61	80.54	88.35
**PlantPathNet (Ours)**	**83.42**	**83.17**	**83.29**	**83.23**	**90.28**

The best values are highlighted in bold.

## 6 Conclusion and future work

This study introduces a methodologically cohesive framework that addresses the specific challenges of leaf-based classification. The innovation stems from the synergistic combination of HSV-based chromatic isolation and cross-layer feature modulation, providing a significant performance leap over standard incremental updates to existing models. The proposed architecture integrates several complementary components, including HSV color space transformation, a Cross-layer Feature Integration Module (CFIM), an efficient channel attention mechanism based on ECANet, and a balanced loss formulation that combines weighted softmax loss with center loss. Extensive experimental evaluations on the PlantVillage dataset demonstrate that *PlantPathNet* consistently outperforms state-of-the-art CNN-based and Vision Transformer–based approaches, achieving an accuracy of 99.57%, precision of 99.52%, recall of 99.54%, F1-score of 99.53%, and an AUROC of 99.84%.

Ablation studies further validate the individual contributions of each proposed component. Specifically, the HSV color space transformation enhances disease-relevant feature representation, CFIM improves feature fusion across multiple network depths, ECANet strengthens channel-wise attention to salient disease characteristics, and the balanced loss function effectively mitigates class imbalance while promoting compact feature distributions. In addition, the proposed model exhibits strong performance in early-stage disease detection and demonstrates superior generalization capability in cross-dataset evaluation scenarios.

Despite these promising results, several directions remain open for future research. First, extending the framework to support multi-disease detection would enable the handling of complex cases in which multiple diseases coexist on a single plant. Second, incorporating explainable artificial intelligence techniques could improve the interpretability of model predictions and provide farmers with actionable insights. Third, further optimization for mobile and edge deployment would facilitate real-time, in-field disease diagnosis without reliance on internet connectivity. Moreover, integrating temporal information could enable monitoring of disease progression and prediction of future spread patterns. The fusion of image data with additional sensing modalities, such as hyperspectral or thermal imaging, also represents a promising avenue for more comprehensive disease assessment. Finally, extending the model to estimate disease severity levels would support more precise and timely treatment recommendations.

Overall, these future research directions have the potential to further enhance the practicality and impact of deep learning–based plant disease diagnosis systems, contributing to sustainable agricultural practices and global food security.

## Data Availability

The original contributions presented in the study are included in the article/supplementary material. Further inquiries can be directed to the corresponding author.
